# A Rare Case of Pancreatic Endometriosis Masquerading as Pancreatic Mucinous Neoplasm

**DOI:** 10.1155/2021/5570290

**Published:** 2021-04-23

**Authors:** Barton Huang, Annie Mooser, Danielle Carpenter, Grace Montenegro, Carrie Luu

**Affiliations:** ^1^Saint Louis University School of Medicine, St. Louis, MO, USA; ^2^SSM Health Saint Louis University, Department of Surgery, St. Louis, MO, USA; ^3^SSM Health Saint Louis University, Department of Pathology, St. Louis, MO, USA

## Abstract

Endometriosis is a relatively common condition among women, and pancreatic endometriosis has been reported on rare occasions. Such pancreatic lesions are difficult to diagnose and distinguish from other cystic lesions of the pancreas preoperatively. This report describes a case of pancreatic endometriosis in a 51-year-old female patient. Imaging demonstrated an enlarging cyst with findings concerning for a mucinous neoplasm. The patient underwent robotic distal pancreatectomy and splenectomy. Histopathology revealed an endometriotic cyst. Pancreatic endometriosis can be difficult to distinguish from other lesions of the pancreas. Surgical resection should be undertaken in cases where malignancy is suspected.

## 1. Introduction

Endometriosis occurs when endometrial cells present outside the uterus, commonly in locations in the pelvis near the uterus, ovaries, and fallopian tubes. It affects up to 10% of women of reproductive age, and 20-25% of cases are asymptomatic [1]. Extrapelvic endometriosis can occur and can present with symptoms such as cyclic pain, bleeding, and a palpable mass [2]. When these lesions are found in the pancreas, the differential includes serous and mucinous neoplasms of the pancreas. Diagnostic investigations, including computed tomography (CT), magnetic resonance imaging (MRI), and endoscopic ultrasound (EUS), are helpful, but diagnostic uncertainty exists.

This case report features a patient with a rare case of endometriosis presenting in the pancreas. While the exact prevalence of extrapelvic endometriosis is not currently known [3], there have been few published cases of pancreatic endometriosis [4–6]. In many cases, patients with pancreatic endometriosis undergo surgical resection as the diagnosis is rarely obtained beforehand.

## 2. Case Presentation

A 51-year-old obese Caucasian female presented for surgical evaluation of an enlarging pancreatic body/tail cyst. The cyst was first diagnosed in 2014, where it measured 1.8 cm and, at that time, was thought to be an intraductal papillary mucinous neoplasm (IPMN) due to imaging characteristics including connection with a pancreatic duct side branch and based on cyst fluid analysis. The cyst fluid demonstrated a CEA of 179 ng/mL and amylase of 12,220 Units/L. The cytologic specimen was hypocellular but did not demonstrate any malignancy. Since that time, she had been undergoing surveillance with radiographic imaging and endoscopic ultrasound.

**Figure 1:**
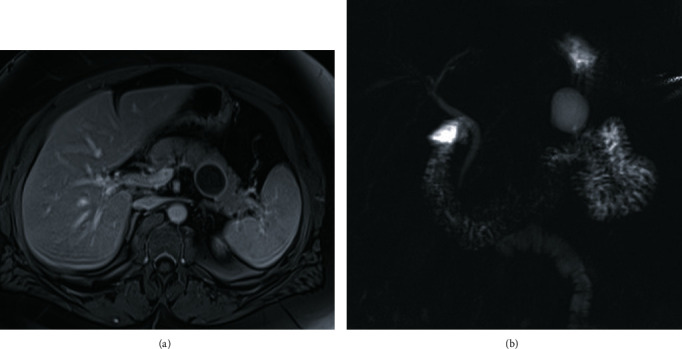
(a) Axial post contrast MRI demonstrates a 3.3 cm pancreatic body/tail cystic lesion without enhancing nodules. (b) MRCP demonstrates a pancreatic body cystic lesion with no discrete connection with the pancreatic duct and a normal pancreatic duct diameter.

On presentation, the patient reported epigastric pain radiating to her back, diarrhea with greasy stools, and anorexia without weight loss. She did not report jaundice or a history of pancreatitis. Her medical history included diabetes and hypothyroidism. Her surgical history was significant for hysterectomy in the distant past for unclear indications; however, she denied symptoms of and surgery for endometriosis. Laboratory findings including cancer antigen 19-9 were unremarkable. EUS demonstrated a likely side branch IPMN measuring 3.5 cm. Magnetic resonance imaging/Magnetic resonance cholangiopancreatography (MRCP) demonstrated a 2.8 × 3.2 × 3.3 cystic pancreatic body lesion consistent with IPMN without main pancreatic duct dilation or enhancing nodules (Figure 1).

**Figure 2:**
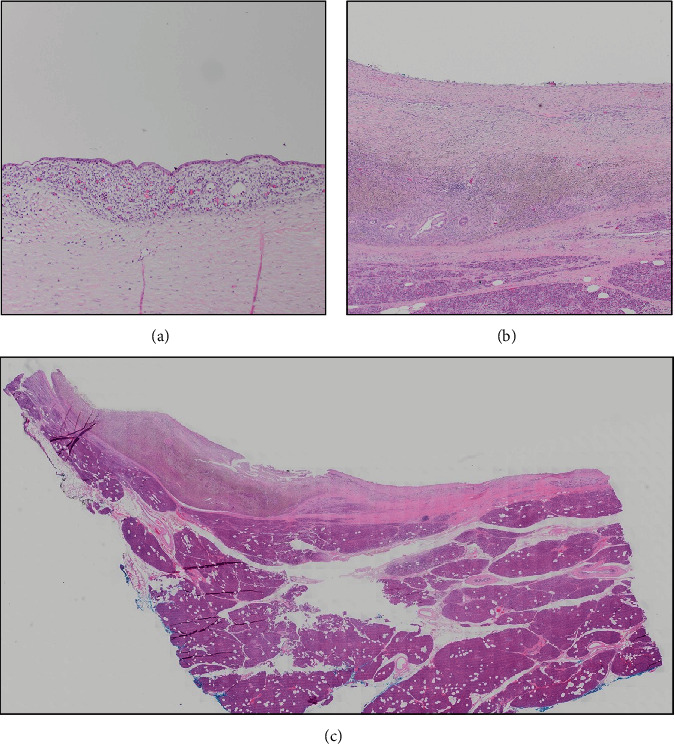
Areas of the cyst are lined by endometrial epithelium and stroma (a), while other areas are denuded but have hemosiderin-laden macrophages underlying the cyst cavity (b). The low power image (c) shows the cyst compressing the surrounding pancreatic parenchyma.

The patient underwent robotic distal pancreatectomy and splenectomy. She was discharged on postoperative day 6 without any complications. Postsplenectomy vaccines were administered before discharge. Final pathology was consistent with endometriotic cyst measuring 3.7 cm. On histopathologic examination, the epithelial lining was bland and without mucinous epithelium commonly seen in intraductal papillary mucinous neoplasm or mucinous cystic neoplasm (MCN); additionally, there was no ovarian type stroma as seen in MCN. Instead, there were areas of endometrial stroma and an overabundance of hemosiderin-laden macrophages along with glandular epithelium overall consistent with endometriosis (Figure 2).

On one-year follow-up, the patient remains well without any symptoms. She does not have any evidence of recurrence of endometriosis on imaging.

## 3. Discussion

On rare occasions, endometriosis can present as pancreatic cystic lesions. Since pancreatic endometriosis is uncommon, no typical imaging features have been formally established [7]. However, two published cases of pancreatic endometriosis proposed features suggestive of pancreatic endometriosis including hyperintense T1-weighted MRI in the presence of bleeding and hypointense T1-weighted or T2-weighted imaging if bleeding is absent [7, 8]. Of these two cases, only one had considered endometriosis on the preoperative differential [8]. Otherwise, the most common presumptive diagnosis included more common pancreatic cystic lesions, such as pseudocysts and serous or mucinous neoplasms.

Pancreatic cystic lesions have been increasingly found due to the rising use of CT and MRI. These incidental pancreatic lesions are found in up to 20% of MRI scans and can portend an increased risk of malignancy [9]. Workup of pancreatic lesions usually involves a multimodal approach including radiography, endoscopic ultrasound, and laboratory studies. However, pancreatic cysts remain difficult to diagnose. For example, studies have shown that the preoperative imaging diagnostic accuracy can range from 68% to 86% [9, 10]. Laboratory studies can also be inconclusive. Elevated cyst fluid CEA levels can indicate MCN, but various studies have shown differing cutoff values, from 109.9 mg/mL up to 800 mg/mL [11, 12]. Likewise, connection with pancreatic duct can indicate an IPMN, though in this case EUS did suggest a connection with a branch duct with was not evident on pathology.

This case demonstrates the difficulty of accurately diagnosing pancreatic cystic lesions preoperatively and the need to keep a broad differential diagnosis. In addition, biopsy should be considered when the diagnosis is uncertain. While the patient in this study did not undergo biopsy, she did meet indications for surgery due to abdominal pain and enlargement of the lesion, and hence, biopsy was unlikely to affect management. For pancreatic lesions, unnecessary surgery should be avoided, but resection remains the treatment of choice should malignancy be suspected.

## 4. Conclusion

Pancreatic endometriosis, though rare, should be considered in the differential for cystic lesions of the pancreas. Despite comprehensive workup including imaging and endoscopic ultrasound with cyst fluid analysis, preoperative diagnosis remains elusive. However, pancreatic lesions meeting criteria for resection, including risk of malignancy and symptoms, should undergo surgical intervention.
